# TIME HELP FROM ADULT CHILDREN DURING THE COVID-19 PANDEMIC: VARIATION BY PARENTS’ COGNITIVE FUNCTIONING

**DOI:** 10.1093/geroni/igad104.0345

**Published:** 2023-12-21

**Authors:** I-Fen Lin, Emily Wiemers, Janecca Chin, Anna Wiersma Strauss

**Affiliations:** Bowling Green State University, Bowling Green, Ohio, United States; Syracuse University, Syracuse, New York, United States; Bowling Green State University, Bowling Green, Ohio, United States; Syracuse University, Syracuse, New York, United States

## Abstract

Many older adults in the U.S. do not live with adult children but have at least one adult child living nearby. Help from adult children is vital for supporting the health and well-being of older adults. During the COVID-19 pandemic, the need to maintain physical distancing and the fear of infection may cause disruptions in older adults’ activities of daily living but the extent to which nonresident adult children provide help with household tasks is largely unknown. This paper uses data from the 2018 and 2020 Health and Retirement Study (HRS) to assess whether adult children responded to their parents’ pandemic-specific needs by helping them with shopping for groceries, errands, rides, or chores (i.e., time help). Preliminary analysis shows that parents who had trouble buying food (even with the money) were more likely to receive time help from their nonresident adult children. Not only was time help much more common among older adults with children living nearby than older adults without any child living nearby but adult children living nearby also were more responsive to parents’ pandemic-specific needs than children who lived farther away. Additional analysis indicates an overall increase in time help from adult children in response to functional limitations between 2018 and 2020, suggesting that the pandemic may have increased family care more broadly. Because older adults with cognitive limitations may be particularly vulnerable during the pandemic, we will further examine whether pandemic-specific needs and time help from nonresident adult children differed depending on older adults’ cognitive functioning.

